# Effect of Psychosocial, Behavioral, and Disease Characteristics on Health-Related Quality of Life (HRQoL) After Breast Cancer Surgery: A Cross-Sectional Study of a Regional Australian Population

**DOI:** 10.7759/cureus.36054

**Published:** 2023-03-12

**Authors:** Madeline Gillies, Keith Tan, Lakmali Anthony, Francis Miller

**Affiliations:** 1 General Surgery, Goulburn Valley Health, Shepparton, AUS; 2 Surgery, St. Vincent's Hospital Melbourne, Melbourne, AUS; 3 Vascular Surgery, The Northern Hospital, Epping, AUS

**Keywords:** eortc qlq, patient-reported outcomes, breast cancer outcomes, quality of life (qol), health-related quality of life (hrqol)

## Abstract

Background: Increasing long-term breast cancer survivorship has highlighted the importance of patient-reported outcomes such as health-related quality of life (HRQoL) in addition to traditional outcomes that were used to define successful operative management. This study aimed to describe HRQoL in patients who underwent breast cancer resection in a regional Australian setting and identify the psychosocial, demographic, and operative characteristics associated with poor HRQoL.

Methods: Consecutive patients who underwent breast cancer resection between 2015 and 2022 were included. Patients were asked to complete a survey instrument that included validated measures of HRQoL, emotional distress, fear of cancer recurrence (FCR), and social support. Demographic, disease, and operative data were collected from the medical record of the respondents.

Results: Forty-six patients completed the survey (100% female, mean age = 62.68 years). Most HRQoL domains were significantly lower than an Australian reference population. HRQoL was more strongly associated with psychosocial factors (emotional distress, FCR, and social support) but was also associated with socioeconomic status, stage of cancer at presentation, and surgical complications. HRQoL was not related to breast conservation, management of the Axilla, or time since operation.

Conclusion: Long-term changes in HRQoL should be considered during the management and surveillance of breast cancer patients in regional Australia.

## Introduction

Breast cancer is the most commonly diagnosed malignancy in Australian women [1]. Overall five-year survival has increased over the past three decades from 77.3% in 1989 to 91.8% in 2021 [1]. With increasing survivorship, the profound and long-lasting impacts that diagnosis, medical and surgical treatment, have on the patient’s physical, social, and emotional well-being have been called to attention [2-3]. Unsurprisingly then, breast cancer survivors have been shown to have decreased health-related quality of life (HRQoL) even after the treatment is complete [4]. Factors previously associated with HRQoL in breast cancer survivors vary by geographic location and have been shown to include clinical factors (including the stage at diagnosis and adjuvant chemotherapy), symptoms (including pain, insomnia, and fatigue), and psychosocial factors (including body image, anxiety, depression, fear of cancer recurrence (FCR), social support, personality, and dispositional optimism) [4-5]. Particularly relevant to surgical practice is the possible long-term impact on HRQoL related to surgical decision-making; for example, the negative impact of mastectomy on body image and social function or decreased physical function as a result of lymphoedema of the upper limb secondary to extensive axillary dissection [6-7]. Despite increased awareness of these issues in breast cancer care, many studies on HRQoL in breast cancer patients focus on medical oncology and lack data on operative technique and surgical complications [4]. Similarly, the examination of HRQoL and other patient-related outcome measures (PROM) in the surgical literature is behind the medical oncology and cancer nursing literature. The 2019 Breast Quality Audit (BQA) report on surgical outcomes produced by Breast Surgeons of Australia and New Zealand (BreastSurgANZ) included only clinical outcome measures. However, a project has begun to assess the feasibility of including PROM in future audits [8].

The patient-reported outcomes (PRO) are outcomes that, in contrast to traditional clinical outcomes (for example, resection margin or disease-free survival in surgical oncology), are reported by the patient rather than the clinician [9]. The PROM are survey-based instruments used to quantify PROs. PROs are a result of the paradigm shift in medicine from the historical paternalistic model to modern patient-centered care. PROMs were initially designed for use in a research setting in order to assess the tolerability and burden of treatment in cancer trials. More recently, the potential benefits of PROM collection in clinical practice have become apparent. Regular assessment of PROs in the clinical environment has been shown to facilitate patient-doctor communication without increasing the length of consultation, improving patients' ability to engage effectively with treatment decisions, and increasing appropriate referral to important allied health services such as psychologists, social workers, and physiotherapists [10-11]. In addition, PROM data have been shown to predict unplanned readmissions and even mortality which can be used to inform quality improvement and safety initiatives [12-13]. A hindrance to the uptake of PROs in surgical research and practice is a lack of widespread understanding of PRO concepts such as HRQoL [14].

The HRQoL is a PRO commonly used in oncology that attempts to characterize the patient's perception of their disease and treatment [3]. It is a multidimensional concept that integrates the medical and psychosocial components of health and disease. Wilson and Cleary [15] developed a conceptual model for understanding HRQoL, the causative factors, and the relationships between them. In this model, biological factors cause symptoms that cause functional derangements, which are modified by environmental (e.g., geographic location and socioeconomic status) and individual (e.g., personality) characteristics. HRQoL can be thought of as the integration of these biological and functional factors in the context of the patient and their environment, leading to the patient's overall perception of health.

To date, there are limited studies that have examined HRQoL in postoperative breast cancer patients in a regional Australian setting [16-18]. This exploratory cross-sectional study aims to: (1) describe postoperative HRQoL in patients who underwent primary resection in a regional Australian hospital; (2) compare HRQoL in this population with reference values of the Australian general population reference values; (3) describe the prevalence of anxiety, depression, and clinically significant FCR in this population; and (4) identify demographic, psychosocial, disease, and treatment factors associated with a poorer self-reported HRQoL. We hypothesize that HRQoL (mean QLQC - 30 score) in this sample will be significantly lower than the general Australian population. Furthermore, we hypothesize the following associations between explanatory variables and QLQC-30 scores; (1) demographic and health factors: age, geographical remoteness, low socioeconomic status, high BMI, smoking, and a history of mental health issues will be associated with poor HRQoL; (2) cancer and surgical factors: recurrence, metastases, ongoing treatment, mastectomy, axillary lymph node dissection (ALND), and major complications will correlate with poor HRQoL, and increasing time since surgery will correlate positively with HRQoL, and; (3) psychosocial PROMs: high anxiety, depression, FCR, body image, and health literacy scores will have a negative association with HRQoL, while social support and optimism will positively correlate with HRQoL.

## Materials and methods

This was a survey-based cross-sectional study conducted at a single regional Australian public hospital. Patients who had an invasive breast cancer resected in the study hospital in January 2015 and July 2022 were eligible to participate. A database of eligible patients was created by searching the hospital information system using the International Classification of Diseases for Oncology (ICD-O) and Australian Refined Diagnosis Related Group (AR-DRG) codes to identify consecutive breast cancer resections during the specified period. Patients recorded as deceased were excluded, and the remaining patients were emailed an invitation to participate that included a summary in plain language and a consent form that required patients who wished to voluntarily participate to approve the review of their hospital medical record, as well as acknowledge that results can be published in a scientific journal without identifiable patient information. Patients who consented were redirected to the survey instrument. Patients without a recorded email address received a package containing hard copies of the same information. Participants were able to withdraw their consent at any time, with contact information on how to do so provided.

Survey and data collection

Participants were asked to complete a survey designed to evaluate HRQoL, as well as other explanatory psychosocial PROs, consisting of a total of 83 items. HRQoL was measured with the European Organization for Research and Treatment of Cancer Quality of Life Questionnaire (EORTC QLQC-30). A ‘summary score’ was also calculated in accordance with published guidelines. This score summarizes all domains of the QLQC-30 (however this is at the expense of information loss) [19]. Body image was measured using four body image questions taken from the EORTC breast-specific module (BR23) [19]. Anxiety and depression were measured using the Hospital Anxiety and Depression Scale (HADS) [20]. Fear of recurrence was measured using the Cancer Worry Scale (CWS) [21]. The Brief Health Literacy Screening Tool (BRIEF) was used to measure health literacy [22]. Social support was measured with the Duke University Social Support Scale Duke University Functional Social Support Questionnaire-Short Form (DUFSS-5) [23]. Finally, dispositional optimism was measured with the Life Orientation Test-Revised (LOT-R) [24].

A review of the medical records was performed to collect demographic and clinical data. Geographic remoteness and socioeconomic disadvantage were quantified using participant postcode. Participants were assigned a score for each based on two Australian Bureau of Statistics classifications; the socioeconomic indexes for areas (SEIFA), and the Australian statistical geography standard - remoteness areas modified Monash model (ASGS-RA MMM). Alcohol use was recorded along the National Health and Medical Research Council Australian Guidelines to Reduce Health Risks from Drinking Alcohol [25].

Reference sample

The QLQC-30 scores of the study population were compared with published reference data from an Australian population [26].

Sample size

The sample size was calculated a priori using guidelines that have published effect sizes and clinical interpretations of the mean differences in each QLQC-30 subdomain score [27]. To detect 'small' differences in global health, functional subdomains, and symptom scales when α =0.05 and β = 0.2, a sample size of n=30 was required.

Data quality

Data were assessed for inconsistent responses and straight line behavior. Missing data were handled using published guidelines for each PROM, and list-wise deletion was used when guidelines were not available [28-29].

Statistical analysis

Descriptive statistics were used to present demographic, clinical, cancer-specific, and surgery-specific data. Age (years), time since surgery (months), length of hospital stay (days), and BMI (kg/m2) were recorded as continuous variables and presented as a mean and standard deviation; all others were categorical and presented as frequencies and valid per cent. To address aim (1),: the mean and standard deviation for all EORTC QLQC-30 domain scores for females in the 40-49, 50-59, 60-69, and >70 groups, and the summary score for females overall in the Australian reference population were recorded [26, 30]. These scores were used to perform independent t-tests for each age group with the hypothesis that the mean score would be lower in the study population compared to the reference population. Tests were considered statistically significant when p=<0.05. Clinical interpretation of mean difference was assigned according to published guidelines on minimal important differences in EORTC QLQC-30 scores [27]. To address aim (2), descriptive statistics were used to present mean and SD scores for HADS A, HADS D and CWS scores, as well as frequencies of cases meeting severity criteria. These were also compared with age using Spearman's rho. To address aim (3), a bivariate analysis was performed between explanatory variables (demographic, cancer, surgery specific, and surgery specific and psychosocial).

## Results

**Figure 1:**
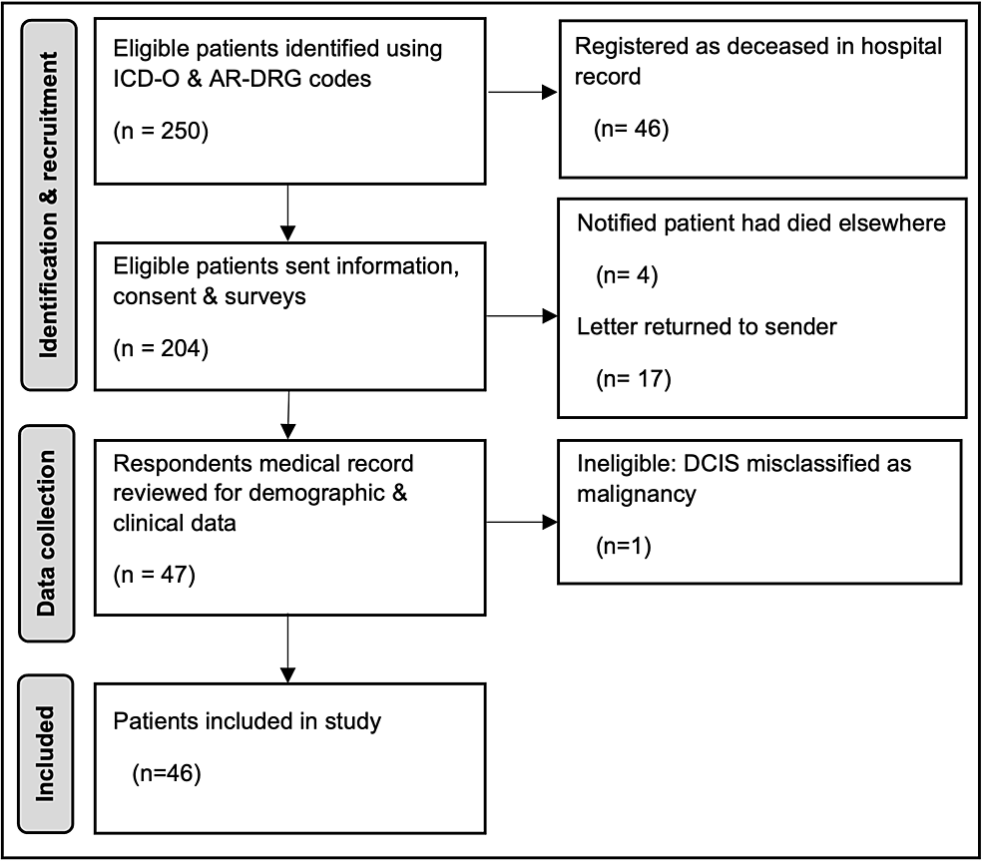
Study methodology and reasons for exclusion. ICD-O, International Classification of Diseases for Oncology; AR-DRG, Australian Refined Diagnosis Related Group

During the period from January 2015 to July 2022, 250 patients underwent breast cancer resection. Forty-six patients were deceased, and 46 eligible completed the survey, so the total participation rate was 22.6%. Reasons for nonparticipation and exclusion at each stage are outlined in Figure 1.

Characteristics of study participants

Demographic and cancer characteristics are summarized in Tables 1-2, respectively. All respondents were female, with a mean age of 62.68 years. Most of the participants lived in small rural towns (56.5%, ASGS-RA MMM5), and an even greater majority lived in so socioeconomically disadvantaged areas (80.4%, SEIFA ≤3). The vast majority of the participants were born in Australia (91.3%) and spoke English at home (97.8%). A small minority of participants were identified as Aboriginal or Torres Strait Islander (4.3%). Most of the participants were retired (55.8%). Participants were overweight (mean BMI 29.8) and non-smokers (60.9%) with minimal alcohol intake (87%). More than a third of the patients had a previous diagnosis of mental illness (39.1%). Regarding their cancers, most were new (93.2%), self-detected (70%), early stage (stage I 37%; stage II 41.3%), and luminal A (59.6%) tumors. Breast conservation surgery (52.2%) with sentinel lymph node biopsy (67.4%), followed by adjuvant radiation (63%) and endocrine therapy (82.6%), were the most common treatment regimens. Some 45.7% of the patients required adjuvant systemic chemotherapy, which was ongoing in a minority of patients (8.9%). The length of hospital stay was often short, with many (38%) day operations. Major complications were uncommon (4.3%), and 34% of patients experienced a minor complication, seroma, or hematoma being the most common (10.9%). There was a large variation in the time since resection ranging from 3 months to 7 years (mean = 43.2 months).

**Table 1:** Sociodemographic characteristics of participants. ASGS-RA MMM, Australian statistical geography standard – remoteness areas modified Monash model; SEIFA, socioeconomic indexes for areas; ASA, American Society of Anesthesiology physical status; NHMRC, National Health and Medical Research Council ; SD, standard deviation; BMI, body mass index

Respondents n(%)	
Age in years (mean, SD)	62.68, 11.2
Sex	
Female	46(100%)
ASGS-RA MMM score	
Metropolitan	2(4.3%)
Large rural town	8(17.4%)
Medium rural town	10(21.7%)
Small rural town	26(56.5%)
Socioeconomic index	
SEIFA score ≤3	37(80.4%)
SEIFA score ≥ 4	9(19.6%)
Language spoken at home	
English	45(97.8%)
Arabic	1(2.2%)
Living arrangement	
Alone	29(64.4%)
With partner	15(33.3%)
Aged care	1(2.2%)
Country of birth	
Australia	42(91.3%)
U.K.	3(6.5%)
Other	1(2.2%)
Indigenous status	
Aboriginal or Torres Strait Islander	2(4.3%)
Employment	
Working	18(41.9%)
Retired	24(55.8%)
Unemployed	1(2.3%)
Marital status	
Single	6(13%)
Married or De facto	27(58.7%)
Divorced	8(17.4%)
Widowed	5(10.9%)
ASA grade	
I	6(13%)
II	24(52.2%)
III	16(34.8%)
Smoking	
Non-smoker	28(60.9%)
Ex-smoker	7(15.2%)
Current smoker	11(23.9%)
BMI (mean, SD)	29.80, 7.20
Alcohol intake	
Within the NHMRC Australian guidelines	40(87%)
More than NHMRC Australian guidelines	6(13%)
Previous mental health diagnosis	18(39.1%)

**Table 2:** Participant tumor and management characteristics. AJCC, American Joint Committee on Cancer; HER2, human epithelial growth factor receptor 2; Luminal A, estrogen and progesterone receptor positive, HER2 negative; Luminal B, estrogen or progesterone receptor positive, HER 2 positive; SLNB, sentinel lymph node biopsy; ALND, axillary lymph node dissection

Respondents n(%)	
Presentation	
Screen-detected	10(25%)
Self-detected	28(70%)
Surveillance	2(5%)
Recurrence	
New diagnosis	41(93.2%)
Recurrent	3(6.8%)
AJCC TNM stage	
Stage I	17(37%)
Stage II	19(41.3%)
Stage III	8(17.4%)
Stage IV	2(4.3%)
Site of metastases	
Bone	2(4.3%)
Liver	1(2.2%)
Lung	1(2.2%)
Molecular type	
Luminal A	32(69.6%)
Luminal B	4(8.7%)
HER2+	4(8.7%)
Triple-negative	6(13%)
Neoadjuvant treatment	
None	36(78.3%)
Chemotherapy	7(15.2%)
Endocrine	3(7%)
Adjuvant treatment	
None	2(4.3%)
Chemotherapy	21(45.7%)
Radiation	29(63%)
Endocrine	38(82.6%)
Current treatment	
Ongoing	4(8.9%)
Ongoing (endocrine only)	29(64.4%)
Treatment complete	12(26.7%)
Breast operation	
Breast conservation	24(52.2%)
Unilateral mastectomy	17(37%)
Bilateral mastectomy	5(10.9%)
Axilla operation	
SLNB	31(67.4%)
ALND	13(28.3%)
None	2(4.3%)
Length of hospital stay in days, mean (SD)	5.11(5.9)
Complications	
None	28(60.9%)
Minor	16(34.8%)
Major	2(4.3%)
Lymphoedema	6(13%)
Re-excision	4(8.7%)
Seroma or hematoma	5(10.9%)
Wound infection	4(8.7%)
Time since surgery in months, mean (SD)	43.24(26.51)

Quality of life

Addressing research aims 1 and 2, Table 3 shows QLQC-30 scores for the study population compared to a reference sample published in 2016. Most HRQoL domains were statistically significantly worse for the study population, particularly for the physical, cognitive, and social function domains for which the differences were considered medium-large. A notable exception was global health which was not significantly different between groups.

**Table 3:** Comparison of HRQoL in the study population and an Australian reference population and a clinical interpretation of the difference. a. Higher scores indicate better health and function; b. higher scores indicate worsening severity of symptoms HRQoL, health-related quality of life; SD, standard deviation; SE, standard error

HRQoL domain	Study population, mean (SD)	Reference population, mean (SD)	Mean difference (SE)	t	df	p	95% CI	Clinical interpretation
Global health^a^	70.6(24.3)	70.1(21.7)	0.52(3.3)	0.16	968	.876	-6.1, 7.1	Trivial
Functional scales^a^								
Physical function	75.5(26.1)	91.7(18.2)	-16.19(2.82)	-5.75	968	<0.001	-21.7, -10.7	Medium
Role function	76.1(30.2)	92.8(22.3)	-16.71(3.43)	-4.87	968	<0.001	-23.5, -10	Small
Emotional function	77.8(22.4)	78.7(24.2)	-0.92(3.64)	-0.25	968	0.811	-8.1, 6.2	-
Cognitive function	77(22.8)	89.3(21.6)	-12.26(3.27)	-3.75	968	<0.001	-18.7, -5.8	Medium
Social function	78.5(28.3)	94.7(22.8)	-16.18(3.49)	-4.64	968	<0.001	-23, -9.3	Large
Symptom scales^b^								
Fatigue	31.4(27.7)	24.2(22.4)	7.2(3.43)	2.1	968	0.036	0.5, 13.9	Small
Nausea and vomiting	6.2(12.4)	3.2(16.5)	2.96(2.47)	1.20	968	0.231	-1.9, 7.8	Trivial
Pain	28.1(29.5)	19.4(26.3)	8.75(4)	2.19	968	0.029	0.9, 16.6	Small
Insomnia	26.8(29.5)	23.2(30.7)	3.61(4.63)	0.78	968	0.436	-5.5, 12.7	Trivial
Appetite loss	24.8(40.1)	7.9(21.8)	16.91(3.47)	4.87	968	<0.001	10.1, 23.7	Medium
Constipation	12.6(23.9)	8.8(22.7)	3.79(3.44)	1.11	968	0.271	-3, 10.5	Trivial
Diarrhea	11.9(23.7)	6(19.4)	5.85(2.96)	1.97	968	0.049	0, 11.7	Small
Financial difficulties	15.2(28.3)	3.3(23.8)	11.85(3.63)	3.27	968	0.001	4.7, 19	Medium
Summary score	79.43(18.3)	81.92(14.9)	2.49(2.37)	1.11	607	0.295	2.9-15.47	-

Anxiety, depression, and fear of recurrence 

Table 4 shows mean HADS anxiety and depression scores by age group, as well as the frequency of mild and moderate to severe symptoms. Overall 23.9% had mild symptoms of anxiety, while 10.9% had moderate to severe symptoms. Depressive symptoms were less common (mild 10.9% and moderate-severe 10.9%). The mean scores for HADS A and HADS D were significantly higher in participants who had a diagnosed mental health disorder prior to surgery (HADS A; p = 0.010, HADS D; p = 0.015). Many participants reported high levels of FCR (68.2%), and most indicated that they were concerned about the possibility that they would need surgery again (70.45%).

**Table 4:** Descriptive statistics for psychosocial variables and spearman correlation with age. ^a^HADS score 8-10; ^b^HADS score 11-15 HADS, hospital anxiety and depression scale

Psychosocial variable	Measure		Age Spearman's rho	p
Anxiety	Mean(SD)	6.2(3.9)	-0.123	0.421
	Mild^a^ n(%)	11(23.9%)		
	Moderate–severe^b^ n(%)	5(10.9%)		
Depression	Mean(SD)	4.7(4.1)	-0.055	0.722
	Mild^a^ n(%)	5(10.9%)		
	Moderate–severe^b^ n(%)	5(10.9%)		
FCR	mean(SD)	16.7(5.2)	-0.308	0.021
	Low–moderate, n(%)	14(31.9%)		
	Severe, n(%)	30(68.2%)		
Body image	Mean(SD)	29.3(35.9)	-0.346	0.021
Social support	Mean(SD)	13(2.6)	0.040	0.397
Optimism	Mean(SD)	14.7(4.7)	-0.300	0.024
Limited health literacy	n(%)	11(24.4%)	0.306	0.020

Social support

Most of the participants were married or in a de facto relationship (58%) and lived with their partner or family (58.7%). Social support was generally high among the participants (mean score = 13, Table 4). The majority of patients indicated that they had love and affection for people who care about them and help with transportation as much as they would like (68.9%, 80%, and 64.4% respectively).

Dispositional optimism 

The mean optimism score was 14.7; this was positively correlated with age (Table 4, p = 0.024). Most patients agreed that they were always optimistic about the future (71.1%) and expected more good things than bad things (89.7%). There was a substantial minority of participants with a more pessimistic disposition, with 37.9% agreeing that they hardly ever expect things to go their way and the same proportion agreeing that if something can go wrong for them, it will.

Body image 

The body image of many participants had been affected, with 48.9% agreeing that they had felt less attractive physically as a result of their disease or treatment, and 46.7% indicating that they had been dissatisfied with their body to some extent. The mean body image score was 29.3; this was significantly correlated with age, with older participants less likely to have poor body image (p = 0.021, Table 4).

Factors associated with HRQoL

Tables 5-7 address research question 4. Demographic and health factors were only sporadically associated with HRQoL (Table 5). Age and BMI did not show an association with any HRQoL domains. Increased geographic remoteness was positively correlated with emotional function. Higher socioeconomic status was associated with increased global health, less fatigue, and less pain. Smoking was correlated with decreased global health, poorer scores in all functional domains, as well as increased fatigue, nausea and vomiting, pain, and financial difficulties. Alcohol use was associated with decreased physical function. History of mental illness was negatively correlated with global health and all functional domains and positively associated with fatigue and appetite loss.

**Table 5:** Spearman correlation coefficients and p-values for the domains of HRQoL and demographic factors and past medical history. a. Higher scores indicate better health and function; b. higher scores indicate worsening severity of symptoms. HRQoL, health-related quality of life; SEIFA, socioeconomic indexes for areas; BMI, body mass index

HRQoL domain	Age	p-value	Geographical remoteness	p-value	SEIFA	p-value	Alcohol use	p-value	History of mental health disorder	p-value	Smoking	p-value	BMI	p-value
Global health^a^	-0.031	0.836	0.009	0.952	0.331	0.025	0.054	0.722	-0.382	0.009	0.548	0.000	0.153	0.311
Functional scales^a^														
Physical function	-0.253	0.090	0.047	0.757	0.034	0.821	0.270	0.069	-0.351	0.017	0.287	0.053	-0.051	0.737
Role function	0.084	0.581	0.082	0.588	0.078	0.607	0.231	0.122	-0.386	0.008	0.440	0.002	-0.120	0.426
Emotional function	0.129	0.398	0.121	0.428	-0.101	0.511	0.100	0.515	-0.451	0.002	0.363	0.014	-0.071	0.645
Cognitive function	0.237	0.117	0.209	0.168	0.211	0.164	0.112	0.463	-0.313	0.037	0.414	0.005	-0.044	0.773
Social function	0.113	0.459	0.111	0.468	-0.012	0.935	0.129	0.398	-0.281	0.061	0.324	0.030	-0.040	0.792
Symptom scales^b^														
Fatigue	-0.035	0.817	-0.060	0.691	-0.264	0.076	-0.133	0.377	0.503	0.000	-0.485	0.001	0.088	0.560
Nausea vomiting	-0.061	0.686	-0.097	0.520	-0.129	0.392	-0.081	0.591	0.187	0.214	-0.306	0.039	0.001	0.997
Pain	0.013	0.933	-0.083	0.586	-0.256	0.090	-0.119	0.435	0.245	0.105	-0.448	0.002	0.069	0.651
Insomnia	-0.087	0.564	-0.022	0.886	0.117	0.437	-0.210	0.161	0.159	0.290	-0.135	0.372	0.029	0.850
Constipation	-0.052	0.733	-0.008	0.960	-0.163	0.283	-0.082	0.592	-0.112	0.466	-0.140	0.360	0.197	0.195
Diarrhea	0.089	0.562	-0.102	0.504	-0.242	0.109	-0.101	0.510	0.171	0.262	-0.205	0.178	0.027	0.862
Loss of appetite	-0.057	0.708	0.133	0.385	-0.127	0.405	-0.235	0.120	0.317	0.034	-0.356	0.016	-0.082	0.593
Financial difficulties	-0.178	0.249	0.031	0.840	-0.194	0.208	-0.006	0.968	0.217	0.158	-0.516	0.000	-0.016	0.918

**Table 6:** Spearman correlation coefficients and p-values for the domains of HRQoL and cancer-specific factors. a. Higher scores indicate better health and function. b. Higher scores indicate worsening severity of symptoms HRQoL, health-related quality of life

HRQoL domain	Stage	p-value	Recurrence	p-value	Ongoing adjuvant	p-value	Breast operation	p-value	Axilla operation	p-value	Complications	p-value	Time since surgery	p-value
Global health^a^	-0.134	0.374	0.025	0.872	-0.303	0.041	0.041	0.785	0.064	0.821	0.278	0.061	0.125	0.407
Functional scales^a^														
Physical function	-0.207	0.168	-0.032	0.835	-0.384	0.008	-0.121	0.423	0.034	0.607	0.178	0.237	0.048	0.753
Role function	-0.206	0.171	0.004	0.981	-0.398	0.006	0.052	0.732	0.078	0.511	0.233	0.120	0.179	0.233
Emotional function	-0.269	0.074	-0.019	0.906	-0.006	0.970	0.097	0.525	-0.101	0.164	0.208	0.171	-0.028	0.858
Cognitive function	-0.002	0.989	-0.221	0.154	-0.145	0.343	0.059	0.703	0.211	0.935	0.252	0.015	0.046	0.764
Social function	-0.341	0.022	-0.020	0.900	-0.022	0.885	-0.037	0.811	-0.012	0.076	0.094	0.540	0.066	0.667
Symptom scales^b^														
Fatigue	0.198	0.188	0.155	0.316	0.260	0.081	-0.145	0.337	-0.264	0.392	-0.176	0.242	-0.005	0.971
Nausea vomiting	0.088	0.562	0.028	0.856	0.049	0.748	-0.015	0.919	-0.129	0.090	-0.266	0.018	0.091	0.548
Pain	0.200	0.189	-0.011	0.942	0.465	0.001	-0.039	0.800	-0.256	0.437	-0.152	0.320	-0.141	0.356
Insomnia	0.178	0.237	0.241	0.115	0.246	0.100	0.135	0.373	0.117	0.283	-0.059	0.695	-0.245	0.101
Constipation	-0.113	0.458	0.061	0.699	0.106	0.487	-0.116	0.448	-0.163	0.109	0.077	0.617	-0.143	0.348
Diarrhea	0.048	0.755	0.248	0.109	0.280	0.062	0.084	0.583	-0.242	0.405	-0.068	0.656	-0.019	0.901
Loss of appetite	0.171	0.262	0.039	0.806	0.277	0.066	-0.151	0.321	-0.127	0.208	-0.129	0.397	-0.239	0.114
Financial difficulties	0.160	0.299	0.300	0.053	0.217	0.157	-0.115	0.456	-0.194	0.672	0.055	0.725	-0.194	0.206

**Table 7:** Spearman correlation coefficients and p-values for the domains of HRQoL and patient-reported outcomes. a. Higher scores indicate better health and function. b. Higher scores indicate worsening severity of symptoms. HRQoL, health-related quality of life; HADS, hospital anxiety and depression scale; FCR, fear of cancer recurrence

HRQoL domain	HADS anxiety	p-value	HADS depression	p-value	FCR	p-value	Social support	p-value	Optimism	p-value	Health literacy	p-value	Body image	p-value
Global health^a^	-0.391	0.008	-0.635	0.000	-0.391	0.009	0.549	0.000	0.292	0.054	-0.191	0.209	-0.446	0.002
Functional scales^a^														
Physical function	-0.458	0.002	-0.567	0.000	-0.279	0.067	0.465	0.001	0.514	0.000	-0.234	0.123	-0.365	0.015
Role function	-0.491	0.001	-0.560	0.000	-0.430	0.004	0.501	0.001	0.335	0.026	-0.143	0.350	-0.330	0.029
Emotional function	-0.716	0.000	-0.504	0.000	-0.686	0.000	0.250	0.102	0.283	0.063	-0.153	0.321	-0.400	0.007
Cognitive function	-0.326	0.031	-0.571	0.000	-0.313	0.039	0.387	0.009	0.115	0.457	0.017	0.912	-0.444	0.003
Social function	-0.318	0.035	-0.497	0.001	-0.391	0.009	0.351	0.019	0.225	0.142	-0.284	0.062	-0.421	0.004
Symptom scales^b^														
Fatigue	0.659	0.000	0.679	0.000	0.431	0.004	-0.503	0.000	-0.391	0.009	0.097	0.526	0.520	0.000
Nausea vomiting	0.432	0.003	0.377	0.011	0.475	0.001	-0.262	0.086	-0.197	0.199	0.050	0.743	0.202	0.188
Pain	0.494	0.001	0.495	0.001	0.289	0.060	-0.367	0.016	-0.182	0.237	0.082	0.592	0.490	0.001
Insomnia	0.335	0.025	0.140	0.359	0.193	0.210	-0.154	0.320	-0.265	0.082	-0.006	0.966	0.250	0.102
Constipation	0.261	0.087	0.186	0.227	0.273	0.076	-0.124	0.428	-0.083	0.590	-0.160	0.295	0.327	0.030
Diarrhea	0.215	0.161	0.456	0.002	0.201	0.192	-0.337	0.025	-0.437	0.003	0.026	0.865	0.089	0.566
Loss of appetite	0.328	0.030	0.143	0.354	0.294	0.056	-0.175	0.263	-0.148	0.336	0.023	0.880	0.043	0.783
Financial difficulties	0.455	0.002	0.355	0.019	0.327	0.032	-0.197	0.206	-0.226	0.140	0.022	0.887	0.143	0.353

Similarly, cancer and surgical factors that were hypothesized to be associated with HRQoL showed only some associations with HRQoL (shown in Table 6). Stage was the most significantly associated, showing a positive correlation with all functional domains and all symptom domains, except constipation. Ongoing adjuvant therapy was associated with poorer global health, physical function, role function, fatigue, pain, insomnia, diarrhoea, and appetite loss. Recurrence was only associated with financial difficulties. Neither type of operation nor time since surgery were correlated with HRQoL.

In contrast, most of the psychosocial PROs hypothesized to be associated with HRQoL were significant correlates of many HRQoL domains (shown in Table 7). Symptoms of anxiety, depression, and FCR were statistically significantly negatively associated with global health and all functional domains and positively associated with many HRQoL symptom scales. Similarly, perceived social support was significantly positively associated with global health and all functional domains, with the exception of emotional function, and negatively associated with symptoms of fatigue, nausea, vomiting, and pain. Dispositional optimism was positively correlated with global health, physical function, role function, and emotional function and negatively associated with symptoms of fatigue and insomnia. Poor body image was a significant correlate between global health and all functional domains.

## Discussion

We hypothesized that the study population would have a poorer HRQoL than the general Australian population. This was largely the case, particularly in the HRQoL functional domains, which were all significantly lower in the study population and all had medium to large clinical importance. The hypothesis that demographic factors would correlate with HRQoL was found to be true primarily for smoking and a history of mental illness. Of the cancer and surgical factors hypothesized to be associated with HRQoL, stage and ongoing adjuvant therapy were the strongest correlates of HRQoL. All psychosocial PROs measured showed some association with HRQoL, particularly anxiety, depression, FCR, and social support.

The HRQoL in the study population was found to be lower than in the general Australian population. HRQoL in breast cancer patients has been shown to be poor compared to controls in many other studies [31]. Similarly, HRQoL in regional breast cancer patients has been shown to be poor compared to metropolitan controls, but this has not been shown in Australia [5, 32]. It should be noted that traditional cancer outcomes have been shown to be worse in regional Australian breast cancer patients compared to metropolitan controls [33]. As such, the characterization of HRQoL in patients with postoperative regional breast cancer in this is a new finding. Ongoing adjuvant endocrine, hormone, and radiation therapy has been shown to correlate with decreased HRQoL in breast cancer populations, supporting the findings in the present study [34-35]. Similarly, the advanced stage and the presence of metastases have been found to be correlated with a decrease in HRQoL in previous research [36]. Other studies have found that mastectomy and extensive axillary dissections are associated with a decrease in HRQoL, which was not the case in the study population [6-7, 35]. This could represent different preferences between women in regional and metropolitan areas, the small number of mastectomy performed, or high-quality shared decision making in this sample. Time since surgery did not correlate with HRQoL in this study. Although cross-sectional studies are unable to demonstrate changes over time, other longitudinal studies have previously shown that HRQoL changes in breast cancer patients do not return to baseline level even 10 years after diagnosis [31, 37]. This finding could imply that even after the biological impact and symptoms associated with medical treatment are gone, function, perception of health, and, ultimately, HRQoL are still affected. This can be understood using Wilson and Cleary's 1995 HRQoL model when considering that emotional and environmental factors moderate these domains of HRQoL [15]. This is supported by the finding in this study that psychosocial PROs appeared to be significantly more associated with HRQoL than any of the biological factors. Anxiety, depression, and FCR all had significant negative effects on the perception of global health, function, and symptoms. Conversely, dispositional optimism and social support had positive effects. This has been shown in other research in which psychosocial PROs often have a greater effect on HRQoL in breast cancer than on disease or treatment-specific factors [5]. It is possible that diagnosis and treatment for breast cancer act as a nidus for the development of significant fear, anxiety, and depression that worsen the perception of patients of their health, affect their ability to function and worsen physical symptoms even once the biological cause is gone, and that personality traits (such as dispositional optimism), and logistic support (such as social supports) mediate this effect. More research is warranted to determine the actual effect of these many complicated factors.

Strengths and limitations

This study has several strengths. Firstly, a wide range of PROMs were used to assess the psychosocial, behavioral, and disease characteristics associated with HRQoL. This has enabled a broad overview of the factors associated with poorer HRQoL, which can help inform shared-decision making for surgeons. Secondly, the comparison of study population HRQoL scores with published reference values from an Australian population allowed the calculation of clinical significance rather than statistical significance alone. The first notable limitation of the study is the sample size. Although the study was powered to detect small clinically important differences in QLQC-30 scores, the subgroups within the population used in the bivariate correlation were not large enough to detect these differences. For example, only two participants identified as Aboriginal or Torres Strait Islander in the sample were included. While the proportion of indigenous participants (4.8%) could be considered comparable to the 3.2% of Australians who identify as Aboriginal or Torres Strait Islander and thus sufficient, the small number of patients means that this important subpopulation is unable to detect clinically important differences, leading to type II error. Studies comparing HRQoL between indigenous and non-indigenous Australians are lacking, but people who identify as Aboriginal or Torres Strait Islander are well known to have significantly worse traditional cancer outcomes [38]. As such, it is important to ensure that they are represented in studies, which unfortunately was not the case in the present study. Lastly, the study is limited by the cross-sectional design. This methodology means that cause and effect are not easy to distinguish, and changes in HRQoL over time remain unexamined. However, with the aim of simply describing HRQoL and factors associated with poor HRQoL in a population not well studied without broad declarations of cause and effect or change in practice, the cross-sectional design is adequate. However, future similar studies may benefit from a longitudinal methodology with an initial preoperative baseline HRQoL from which to draw inferences regarding changes over time and comment on incidence rather than simple prevalence of poor HRQoL.

## Conclusions

The HRQoL is an important outcome in surgical oncology. It is affected not only by patients' disease but its treatment, as well as the patient and their personality, emotional state, and physical environment. Understanding HRQoL and awareness of patients particularly vulnerable to a poor outcome should be used to aid the informed consent and shared decision-making process between surgeon and patient.

## References

[1] (2022). AIHW: Cancer data in Australia. https://www.aihw.gov.au/reports/cancer/cancer-data-in-australia/contents/about.

[2] (2022). Breast Cancer Network Australia: state of the nation report. https://www.bcna.org.au/about-us/advocacy/research-reports/state-of-the-nation-report/.

[3] Saunders CM, Stafford L, Hickey M (2022). Surviving and thriving after breast cancer treatment. Med J Aust.

[4] Javan Biparva A, Raoofi S, Rafiei S (2022). Global quality of life in breast cancer: systematic review and meta-analysis. BMJ Support Palliat Care.

[5] Culbertson MG, Bennett K, Kelly CM, Sharp L, Cahir C (2020). The psychosocial determinants of quality of life in breast cancer survivors: a scoping review. BMC Cancer.

[6] Türk KE, Yılmaz M (2018). The effect on quality of life and body image of mastectomy among breast cancer survivors. Eur J Breast Health.

[7] Tang NS, Ramakrishnan A, Shayan R (2021). Quality-of-life outcomes after operative management of primary and secondary lymphoedema: a systematic review. ANZ J Surg.

[8] (2022). BreastSurgANZ Quality Audit Annual Report. https://www.surgeons.org/-/media/Project/RACS/surgeons-org/files/morbidity-audits/BQA_Annual_Report_2019.pdf?rev=645ce9dd0c6844f3bb144c9750843ec2&hash=7F96B063C29CCC83FEB97DA5F0B50155.

[9] (2022). Patient-reported outcome measures: literature review. https://www.safetyandquality.gov.au/publications-and-resources/resource-library/patient-reported-outcome-measures-literature-review.

[10] Greenhalgh J, Gooding K, Gibbons E, Dalkin S, Wright J, Valderas J, Black N (2018). How do patient reported outcome measures (PROMs) support clinician-patient communication and patient care? A realist synthesis. J Patient Rep Outcomes.

[11] (2022). 2020-25 National Health Reform Agreement (NHRA). https://www.health.gov.au/our-work/2020-25-national-health-reform-agreement-nhra.

[12] Basch E, Deal AM, Kris MG (2016). Symptom monitoring with patient-reported outcomes during routine cancer treatment: a randomized controlled trial. J Clin Oncol.

[13] Gotay CC, Kawamoto CT, Bottomley A, Efficace F (2008). The prognostic significance of patient-reported outcomes in cancer clinical trials. J Clin Oncol.

[14] Troidl H (1991). Quality of life: definition, conceptualization and implications —a surgeon's view. Theoret Surg.

[15] Wilson IB, Cleary PD (1995). Linking clinical variables with health-related quality of life. A conceptual model of patient outcomes. JAMA.

[16] DiSipio T, Hayes S, Newman B, Janda M (2009). What determines the health-related quality of life among regional and rural breast cancer survivors?. Aust NZ J Public Health.

[17] Hayes SC, Rye S, Battistutta D, DiSipio T, Newman B (2010). Upper-body morbidity following breast cancer treatment is common, may persist longer-term and adversely influences quality of life. Health Qual Life Outcomes.

[18] Sherman KA, Heard G, Cavanagh KL (2010). Psychological effects and mediators of a group multi-component program for breast cancer survivors. J Behav Med.

[19] Fayers P, Aaronson NK, Bjordal K, Sullivan M (2001). QLQ-C30 Scoring Manual 3rd Edition. European Organisation for Research and Treatment of Cancer, Brussels.

[20] Zigmond AS, Snaith RP (1983). The hospital anxiety and depression scale. Acta Psychiatr Scand.

[21] Custers JA, van den Berg SW, van Laarhoven HW, Bleiker EM, Gielissen MF, Prins JB (2014). The Cancer Worry Scale: detecting fear of recurrence in breast cancer survivors. Cancer Nurs.

[22] Wallace LS, Rogers ES, Roskos SE, Holiday DB, Weiss BD (2006). Brief report: screening items to identify patients with limited health literacy skills. J Gen Intern Med.

[23] Saracino R, Kolva E, Rosenfeld B, Breitbart W (2015). Measuring social support in patients with advanced medical illnesses: an analysis of the Duke-UNC Functional Social Support Questionnaire. Palliat Support Care.

[24] Hinz A, Sander C, Glaesmer H, Brähler E, Zenger M, Hilbert A, Kocalevent RD (2017). Optimism and pessimism in the general population: psychometric properties of the Life Orientation Test (LOT-R). Int J Clin Health Psychol.

[25] Conigrave KM, Ali RL, Armstrong R (2021). Revision of the Australian guidelines to reduce health risks from drinking alcohol. Med J Aust.

[26] Mercieca-Bebber R, Costa DS, Norman R (2019). The EORTC quality of life questionnaire for cancer patients (QLQ-C30): Australian general population reference values. Med J Aust.

[27] Cocks K, King MT, Velikova G, Martyn St-James M, Fayers PM, Brown JM (2011). Evidence-based guidelines for determination of sample size and interpretation of the European Organisation for the Research and Treatment of Cancer Quality of Life Questionnaire Core 30. J Clin Oncol.

[28] Kang H (2013). The prevention and handling of the missing data. Kor J Anesthesiol.

[29] Bell ML, Fairclough DL, Fiero MH, Butow PN (2016). Handling missing items in the Hospital Anxiety and Depression Scale (HADS): a simulation study. BMC Res Notes.

[30] Mercieca-Bebber R, Campbell R, Fullerton DJ (2023). Health-related quality of life of Australians during the 2020 COVID-19 pandemic: a comparison with pre-pandemic data and factors associated with poor outcomes. Qual Life Res.

[31] Maurer T, Thöne K, Obi N, Jung AY, Behrens S, Becher H, Chang-Claude J (2021). Health-related quality of life in a cohort of breast cancer survivors over more than 10 years post-diagnosis and in comparison to a control cohort. Cancers (Basel).

[32] Moss JL, Pinto CN, Mama SK, Rincon M, Kent EE, Yu M, Cronin KA (2021). Rural-urban differences in health-related quality of life: patterns for cancer survivors compared to other older adults. Qual Life Res.

[33] Roder D, Zorbas HM, Kollias J (2014). Analysing risk factors for poorer breast cancer outcomes in residents of lower socioeconomic areas of Australia. Aust Health Rev.

[34] Roine E, Sintonen H, Kellokumpu-Lehtinen PL (2020). Health-related quality of life of breast cancer survivors attending an exercise intervention study: a five-year follow-up. In Vivo.

[35] Park J, Rodriguez JL, O'Brien KM, Nichols HB, Hodgson ME, Weinberg CR, Sandler DP (2021). Health-related quality of life outcomes among breast cancer survivors. Cancer.

[36] Ghislain I, Zikos E, Coens C (2016). Health-related quality of life in locally advanced and metastatic breast cancer: methodological and clinical issues in randomised controlled trials. Lancet Oncol.

[37] Koch L, Jansen L, Herrmann A (2013). Quality of life in long-term breast cancer survivors - a 10-year longitudinal population-based study. Acta Oncol.

[38] Garvey G, Cunningham J, Mayer C, Letendre A, Shaw J, Anderson K, Kelly B (2020). Psychosocial aspects of delivering cancer care to indigenous people: an overview. JCO Glob Oncol.

